# Humanized Mouse Models as a Cellular Platform for Investigating Immune‐Hormonal Crosstalk and Therapeutic Strategies in Menopause

**DOI:** 10.1111/acel.70313

**Published:** 2025-12-10

**Authors:** Nisansala Chandimali, Jaehoon Bae, Sun Hee Cheong, Seon Gyeong Bak, Yeon‐Yong Kim, Ji‐Su Kim, Seung‐Jae Lee

**Affiliations:** ^1^ Functional Biomaterial Research Center Korea Research Institute of Bioscience and Biotechnology (KRIBB) Jeongeup Korea; ^2^ Applied Biological Engineering, KRIBB School of Biotechnology University of Science and Technology Daejeon Korea; ^3^ Functional Food Research Institute Industry‐University Cooperation Foundation, Daegu‐Hanny University Gyeongsan Korea; ^4^ Department of Marine Bio Food Science Chonnam National University Yeosu Korea; ^5^ Primate Resources Center (PRC) Korea Research Institute of Bioscience and Biotechnology (KRIBB) Jeongeup Korea

**Keywords:** estrogen, functional food, humanized mice, immunity, menopause

## Abstract

Menopause is a complex biological transition marked by the decline of ovarian function and estrogen levels, leading to a wide range of physiological and immunological changes in women. Traditional animal models, particularly ovariectomized rodents, have been instrumental in studying menopause; however, they often fail to fully replicate human hormonal and immune responses. Humanized mouse models, which incorporate human immune systems and tissues, represent a promising alternative for bridging this translational gap. This review explores the current applications of humanized mice in disease research and highlights their untapped potential in menopause studies. We discuss the limitations of existing menopause models and propose a novel framework for using humanized mice to investigate estrogen signaling, immune interactions, and functional food interventions. Functional foods such as soy isoflavones, polyphenols, omega‐3 fatty acids, and probiotics have shown beneficial effects on menopausal symptoms in clinical and animal studies, yet their immune‐modulatory mechanisms remain underexplored in human‐relevant models. We advocate for interdisciplinary collaboration to develop and utilize humanized mouse models tailored to menopause research. This integrated approach may offer new insights into the immune‐hormonal landscape of menopause and pave the way for personalized, non‐invasive therapeutic strategies.

## Graphical abstract

Humanized mice offer new insight into immune‐hormonal changes during menopause. Traditional models lack accuracy in replicating human menopause physiology. Functional foods may ease symptoms but need testing in human‐relevant systems. Combining humanized mice with diet research can improve women's health outcomes.

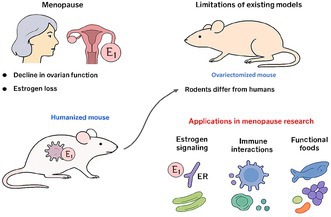

## 1 Introduction

Animal models have long been indispensable in advancing our understanding of human diseases. Among them, humanized mouse models, which are genetically engineered to express human genes, tissues, or immune systems, have emerged as a powerful tool in preclinical research (Tyagi et al. 2020). These models are particularly valuable in studying diseases where species‐specific immune responses play a critical role, such as cancer, autoimmune disorders, and infectious diseases like HIV (Walsh et al. 2017). By bridging the gap between traditional animal models and human clinical trials, humanized mice offer unparalleled insight into human‐specific physiological and pathological mechanisms.

Despite the growing use of humanized mice in various disease contexts, their potential in the field of women's health, particularly menopause, remains underexplored. Menopause, a natural biological transition characterized by the cessation of ovarian function and a sharp decline in estrogen levels, leads to a multitude of physiological changes (Nieto et al. 2025). These include hot flashes, mood disturbances, decreased bone density, cardiovascular risks, and metabolic alterations. While ovariectomized (OVX) rodent models have been commonly employed to mimic menopause, they fall short in replicating the complexity of human immune and hormonal interactions (Baeza et al. 2010; Medina‐Contreras et al. 2020). Furthermore, traditional animal models do not accurately reflect the nuanced metabolic and immune responses seen in postmenopausal women, which is crucial when evaluating potential therapeutic interventions (Chalvon‐Demersay et al. 2017; Diaz Brinton 2012).

This gap underscores the urgent need for more human‐relevant models to study menopause and its systemic effects. A well‐designed humanized mouse model could provide a transformative platform for investigating the interplay between the immune system, hormonal regulation, and metabolic health in postmenopausal conditions. Such models would enable researchers to evaluate therapeutic strategies in a setting that better mirrors the human physiological environment.

In parallel, there is a growing interest in using functional foods and nutraceuticals as natural alternatives to hormone replacement therapy (HRT) for alleviating menopausal symptoms (Meegaswatte et al. 2024). Compounds like phytoestrogens, polyphenols, omega‐3 fatty acids, and fermented products have demonstrated beneficial effects on bone health, mood regulation, and cardiovascular function in both preclinical and clinical studies (De Franciscis et al. 2019; Méndez and Medina 2021). However, the application of these interventions has not yet been rigorously tested in humanized models, particularly not in the context of menopause.

**FIGURE 1:**
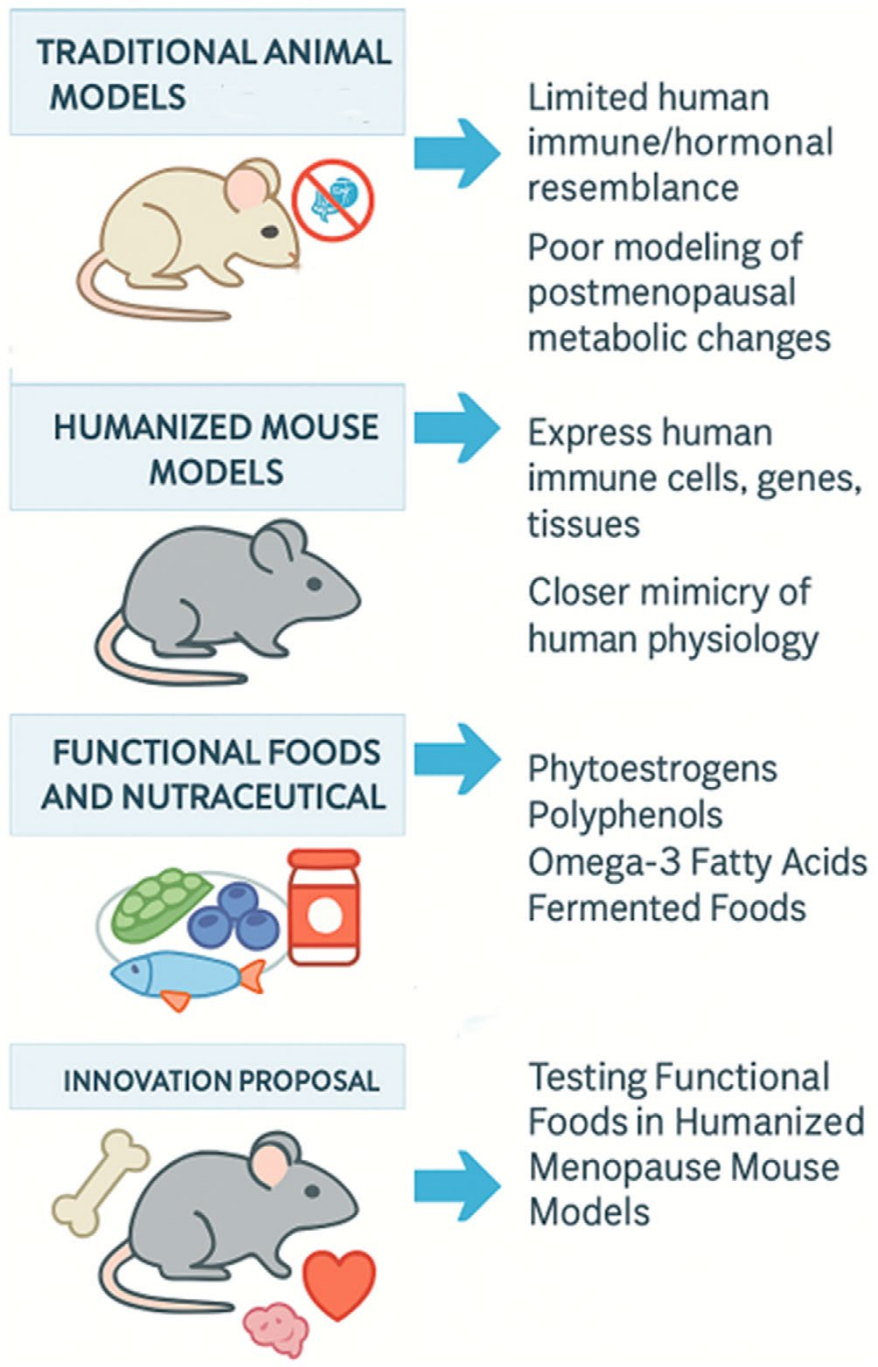
Humanized mouse models as a novel platform for menopause research. Humanized mice, genetically engineered to express human genes, tissues, or immune systems, are widely used in cancer and autoimmune research but remain underutilized in menopause studies. These models could provide a translational platform to study menopause‐induced hormonal, immune, and metabolic changes and to assess the impact of functional food interventions on human‐like physiological responses.

In this review, we aim to explore the current landscape of humanized mouse models used in disease research, identify the existing research gap in applying these models to menopause, and propose an innovative framework to use humanized mice for assessing the efficacy of functional food interventions in menopause‐related conditions. Through this integrated approach, we hope to inspire new directions in translational research that better reflect the complexities of human physiology (Figure 1).

## 2 Humanized Mouse Models: Current Applications in Disease Research

**FIGURE 2:**
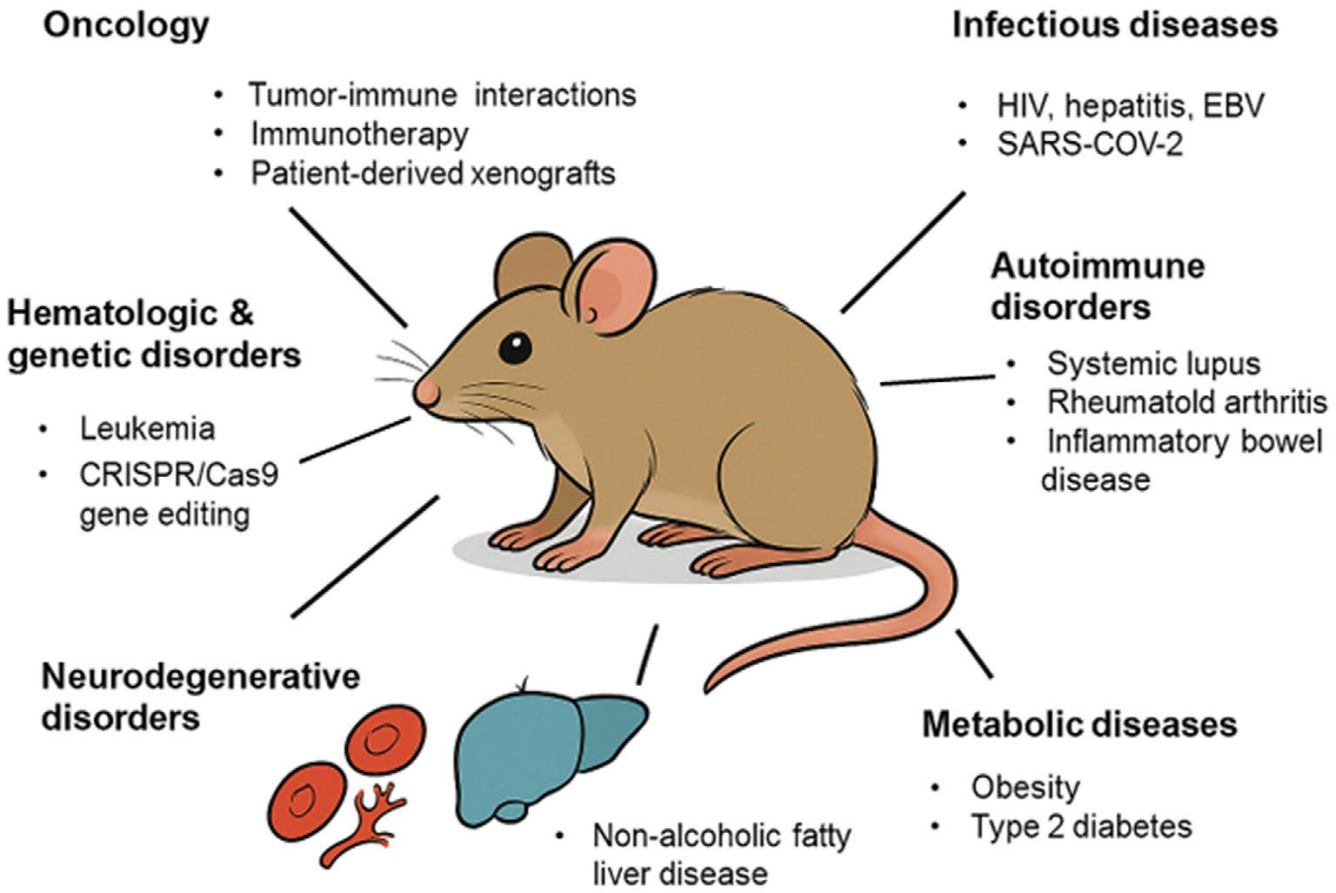
Applications of humanized mouse models in disease research. Humanized mice are extensively used in studying oncology, infectious diseases, autoimmune disorders, hematologic and genetic disorders, neurodegenerative diseases, and metabolic disorders by replicating human‐specific physiological and immune responses.

Humanized mouse models have become indispensable tools in biomedical research due to their ability to simulate human‐specific physiological responses within a controlled and manipulable in vivo environment (Walsh et al. 2017) (Figure 2). These models are genetically engineered to express human genes or to host human cells, tissues, or immune systems, offering a more translationally relevant platform than traditional murine models (Chuprin et al. 2023). The two primary types of humanized models, genetically modified mice and immune system humanized mice, have both opened doors for studying diseases that do not naturally manifest or progress in mice, including a wide array of infectious, autoimmune, oncologic, and metabolic diseases (Brehm et al. 2014; Yang et al. 2024).

In oncology, humanized mice are routinely used to evaluate tumor‐immune interactions and immunotherapeutic interventions. Models engrafted with human immune systems can simulate T cell activation, checkpoint inhibition, and tumor response to various treatments, such as CAR‐T cell therapy or monoclonal antibodies (Chuprin et al. 2023; Park et al. 2025). Patient‐derived xenograft (PDX) models, when combined with a human immune system, allow for personalized medicine approaches, providing preclinical insights into individual patient responses and resistance mechanisms. The precision and adaptability of these models have accelerated the development of new cancer therapies and biomarkers (Liu and Yang 2025; Liu et al. 2023).

Infectious disease research has perhaps benefited the most from these platforms. Humanized mice have enabled the study of viruses such as HIV, hepatitis B and C, Epstein–Barr virus, and human cytomegalovirus, pathogens that do not infect murine cells (Dash et al. 2021). For example, human immune system‐engrafted mice allow for the study of HIV replication, latency, and therapeutic intervention, while liver‐humanized mice facilitate research on hepatotropic viruses (Lai and Chen 2018). More recently, humanized ACE2‐expressing mice have been instrumental in the evaluation of SARS‐CoV‐2 pathogenesis, immune responses, and vaccine efficacy, reflecting their timely relevance and adaptability during emerging pandemics (Jiang et al. 2020).

Autoimmune and inflammatory disorders, such as systemic lupus erythematosus, rheumatoid arthritis, inflammatory bowel disease, and multiple sclerosis, have also been modeled in humanized systems. These diseases are characterized by complex immunopathology involving human‐specific immune responses that cannot be fully replicated in traditional mouse models (Chen, Liao, et al. 2022; Schinnerling et al. 2019). Humanized mice reconstituted with human hematopoietic stem cells (HSC) have been utilized to study the roles of T regulatory cells, cytokine signaling, and immune cell infiltration, offering key insights into disease mechanisms and immunomodulatory therapy development (Negi et al. 2021).

Beyond immune‐related disorders, humanized mice have been employed in the study of hematologic malignancies and genetic conditions, particularly those affecting the blood and bone marrow (Tyagi et al. 2020). Engraftment of patient‐derived hematopoietic cells into immunodeficient mice allows for detailed investigations into leukemia progression, hematopoietic differentiation, and in vivo testing of gene editing tools like CRISPR/Cas9 for diseases such as sickle cell anemia and β‐thalassemia. These models are essential for understanding therapeutic reprogramming and assessing long‐term safety and efficacy of genome‐editing technologies (Samuelson et al. 2021).

Although still an emerging area, neurodegenerative and psychiatric disorder research is beginning to leverage humanized models (Tello et al. 2022). Studies investigating Alzheimer's disease, Parkinson's disease, and neuroinflammatory conditions are using models engrafted with human microglial precursors to explore neuroimmune crosstalk and inflammation‐induced neurodegeneration (Balestri et al. 2024). While these applications are in the early stages, they suggest the potential for uncovering novel mechanisms and therapeutic targets within the central nervous system.

Metabolic diseases and lifestyle‐related disorders such as obesity, type 2 diabetes, and non‐alcoholic fatty liver disease have also begun to be explored using humanized mice. These conditions are intricately linked to immune dysregulation and chronic low‐grade inflammation (Saxena et al. 2022). Humanized models offer the advantage of studying how the human immune system interacts with metabolic tissues under different dietary interventions or drug treatments. Though still limited, these early studies highlight the value of integrating immunometabolic dynamics in preclinical research (Kisoh et al. 2021).

Importantly, Human Immune System (HIS) mice, which are generated by engrafting immunodeficient strains such as NSG, NOG, or BRG with human hematopoietic stem cells, exhibit considerable variability that can affect research outcomes. Factors such as genetic background differences, source and type of human cells, suboptimal lymph node and myeloid cell development, microbiota composition, cytokine requirements, and post‐engraftment time can all contribute to inconsistent results. Moreover, humanized mice have shorter lifespans and distinct health profiles compared to standard murine models, which may limit long‐term or nutritional intervention studies. Therefore, standardization of experimental design, housing conditions, and detailed reporting of model characteristics are essential to improve reproducibility and translational reliability. When applied to menopause research, it will also be critical to define how complex symptoms, such as bone loss, cognitive decline, vasomotor instability, and immune aging, can be evaluated in humanized mice relative to conventional OVX models. HIS mice could be monitored through measurable physiological and molecular parameters to serve as proxies for human menopausal symptoms, enabling a more systematic comparison across models.

Despite the vast contributions of humanized mice to disease modeling, one notably underexplored field is that of reproductive and hormonal health, particularly female reproductive aging and menopause. Most current menopause models rely on surgical removal of ovaries in mice or rats, which mimic some symptoms but lack the complexity of human hormonal and immune interplay (Diaz Brinton 2012). The immune system plays a critical role in menopausal transition and related conditions such as osteoporosis, cardiovascular dysfunction, and metabolic syndrome (Camon et al. 2024). However, few if any studies have investigated these phenomena using a fully humanized in vivo platform. This gap presents an exciting opportunity to advance menopause research by integrating a human immune system into murine models, allowing for a deeper understanding of estrogen withdrawal, immune senescence, and systemic inflammation in postmenopausal physiology. Such an approach would offer novel perspectives not only in pathophysiological research but also in therapeutic validation, especially in testing natural compounds and functional foods tailored for women's health.

In summary, the application of humanized mouse models has significantly broadened our understanding of human diseases, providing more predictive and translational data across multiple disciplines. Yet, their integration into women's health research, particularly menopause, remains largely untapped. Incorporating HIS mice considerations, along with standardized assessment criteria for menopausal symptoms, will be essential for improving experimental reproducibility and translatability. Exploring this intersection may not only bridge current research gaps but also support the development of more targeted, holistic treatment strategies, including the use of functional food interventions in the aging female population.

## 3 Humanized Mouse Models and Women's Health

**FIGURE 3:**
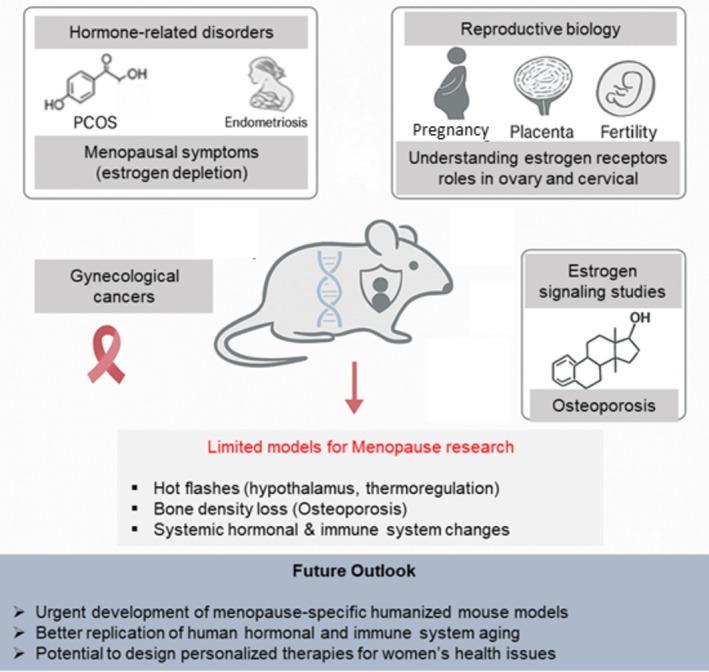
Humanized mouse models in women's health research. Possible applications of humanized mice in studying hormone‐related disorders such as polycystic ovary syndrome (PCOS), reproductive biology, gynecological cancers, and estrogen signaling and current limitations in modeling menopause‐related conditions and emphasize the need for menopause‐specific models to better replicate human hormonal and immune aging.

The exploration of humanized mouse models in women's health is a relatively new and exciting frontier in biomedical research, particularly when it comes to hormone‐related conditions, reproductive biology, gynecological cancers, and estrogen signaling studies (Figure 3).

Humanized mice, which are genetically modified to harbor a functional human immune system or, in some cases, humanized organs, offer unparalleled opportunities to mimic human physiology and pathophysiology more closely than traditional animal models (Shultz et al. 2012). This has proven particularly valuable in studying diseases and conditions that disproportionately affect women, such as hormone‐related disorders, menopause, and various forms of cancer, which may have different biological mechanisms in women compared to men (Fu et al. 2014). However, while humanized mice have been increasingly utilized in research, the application of these models to menopause and related mechanisms, such as estrogen depletion, bone density loss, and hot flashes, remains largely unexplored. Nevertheless, the promise of these models lies in their potential to fill existing gaps in our understanding and develop more effective treatments.

Hormone‐related conditions, such as polycystic ovary syndrome (PCOS), endometriosis, and menopausal symptoms, are prevalent in women and are known to have a significant impact on their quality of life (Sadeghi 2022). Traditional animal models, particularly those based on rodents, have been invaluable in understanding the underlying mechanisms of these conditions. However, they have limitations in recapitulating the complexities of human disease, particularly regarding the influence of hormones on immune responses, metabolic processes, and aging (He et al. 2022; Malvezzi et al. 2020). Humanized mouse models provide a promising alternative, as they can be engineered to have human‐like immune responses and hormone signaling pathways, offering a more accurate representation of these conditions (Shultz et al. 2012). For instance, humanized mice could be used to investigate how hormonal fluctuations during menopause influence immune system function, inflammation, and bone density, key issues for postmenopausal women.

In the context of reproductive biology, humanized mice have made significant strides in modeling human pregnancy, labor, and fertility. While traditional mouse models have been essential for understanding reproductive physiology, humanized models are being increasingly used to replicate human‐specific conditions that may not be effectively studied in standard models. These include immune responses to pregnancy and the placental microenvironment, as well as the mechanisms underlying fertility and infertility (Dong et al. 2024). Humanized mice have already been employed to explore immune tolerance during pregnancy, particularly the maternal‐fetal interface, which is crucial for a healthy pregnancy (Dong et al. 2024). As reproductive health continues to evolve, humanized models could offer critical insights into the mechanisms behind fertility decline and menopause, including the hormonal shifts that lead to ovarian aging and reduced fertility.

The study of gynecological cancers, such as ovarian, cervical, and endometrial cancers, is another critical area where humanized mice models are making a substantial impact (Lõhmussaar et al. 2020). Gynecological cancers have unique characteristics in women, including estrogen receptor (ER) expression, immune system interactions, and tumor microenvironment dynamics (Kozieł and Piastowska‐Ciesielska 2023). Humanized mouse models, particularly those that have been engrafted with human tumor tissue, are invaluable in studying the immune responses to cancer therapies and the role of estrogen in cancer progression (Chen et al. 2023). These models can help researchers test potential cancer treatments that target ERs or immune cells, offering more reliable predictions for human treatment outcomes. However, in the case of menopause, where hormonal withdrawal occurs, it would be particularly beneficial to develop humanized mouse models that can better replicate the hormonal changes and related symptoms experienced by postmenopausal women. In this regard, a potential gap exists; there has been limited research focusing on menopause‐related conditions using humanized mice, despite their great potential in advancing this area of study.

One area where humanized mouse models have gained traction is in estrogen signaling studies (Scherer et al. 2021). Estrogen, a crucial hormone in women's health, is involved in a variety of biological processes, including reproductive health, bone density regulation, and cardiovascular health. Estrogen signaling is also implicated in several diseases, including breast cancer, osteoporosis, and autoimmune disorders (Chen, Li, and Ou‐Yang 2022). Humanized mice models, particularly those with human ERs, provide an excellent tool for understanding the intricate mechanisms of estrogen signaling in a more human‐like context. These models are essential for studying how estrogen influences tissue‐specific responses and how estrogen deprivation, such as in menopause, affects the body. For example, research on osteoporosis, a common condition in postmenopausal women, has been significantly enhanced through the use of ER‐deficient mice or humanized models that incorporate human‐like hormonal regulation (Zhao et al. 2018; Ziemian et al. 2021). This has allowed for the investigation of new therapeutic approaches, including estrogen replacement therapies and alternative treatments.

Despite these advances, a critical gap exists in the specific application of humanized mice models to study menopause and menopause‐related mechanisms. Estrogen depletion, bone density loss, hot flashes, and other common symptoms of menopause have been studied in animal models, but these studies typically use standard rodent models rather than humanized versions (Camon et al. 2024). While these traditional models provide valuable information, they do not fully replicate the complex hormonal changes and immune system interactions that occur in postmenopausal women (Koh et al. 2024). The lack of humanized models in this area is a significant limitation, as menopause involves a range of systemic changes that are not well captured by standard mouse models, including changes in immune function, hormonal regulation, and aging processes.

Moreover, hot flashes, one of the hallmark symptoms of menopause, have yet to be studied in humanized mouse models. Hot flashes, which are thought to be triggered by fluctuations in estrogen levels, represent a unique challenge in menopause research. They are characterized by sudden feelings of warmth, often accompanied by sweating and rapid heartbeats, and are believed to be linked to changes in the hypothalamus and thermoregulation processes (Freedman 2014). While hot flashes are widely recognized in human populations, their underlying mechanisms are not fully understood, and traditional animal models have been limited in replicating these symptoms. Humanized mice models could offer a more accurate model for studying hot flashes by incorporating human‐specific signaling pathways involved in thermoregulation and hormonal fluctuations.

Another critical gap is the role of humanized mice in studying bone density loss during menopause. Estrogen plays a vital role in maintaining bone health, and its depletion during menopause leads to increased bone resorption, ultimately resulting in osteoporosis (Weitzmann and Pacifici 2006). Current models of osteoporosis rely on animals that are estrogen‐deficient or ovariectomized, but these models may not adequately represent the complex hormonal and immune interactions that occur in postmenopausal women (Kan 2013). Humanized mouse models that incorporate human‐like estrogen signaling could be used to study the precise mechanisms by which estrogen depletion affects bone remodeling and to test potential therapies more effectively. Such models could also help identify novel therapeutic targets for preventing or reversing bone density loss, a crucial issue for postmenopausal women.

In conclusion, while humanized mouse models have made significant strides in advancing research on hormone‐related conditions, reproductive biology, and gynecological cancers, their application to menopause and menopause‐related mechanisms is still in its infancy. There is an urgent need for the development of humanized models that can better replicate the hormonal fluctuations, immune responses, and systemic changes associated with menopause. By bridging this gap, humanized mice could become an invaluable tool for studying menopause and developing targeted therapies to alleviate its symptoms, improve bone health, and mitigate the risk of chronic diseases associated with estrogen depletion. As research in this area progresses, humanized mice models have the potential to revolutionize our understanding of menopause and pave the way for more effective, personalized treatments for women's health.

## 4 Proposed Use of Humanized Mouse Models in Menopause Research

The exploration of menopause through animal models has long been constrained by the limitations of traditional systems, particularly in replicating the nuanced hormonal and immune transitions observed in women (Diaz Brinton 2012). As the previous section outlined, OVX rodents have provided foundational insights, but they fall short in modeling the slow and complex hormonal decline and its interplay with human‐specific immune functions. This growing recognition of the need for more refined tools has led to a compelling proposition: the development of humanized mouse models that accurately reflect menopausal physiology. These advanced models could revolutionize our understanding of menopause and its associated health risks by allowing scientists to investigate immune‐hormonal interactions in a more human‐relevant context.

A humanized mouse model designed specifically for menopause research would need to incorporate several key features to ensure physiological relevance. Most critically, it would involve the integration of a functional human immune system and human‐like hormonal signaling pathways. To date, humanized mice are typically generated by engrafting immunodeficient mice, such as the NOD‐scid IL2Rγnull (NSG) strain, with human HSCs derived from sources like cord blood or peripheral blood (Xia et al. 2019). This allows the mouse to develop a human‐like immune system, capable of mimicking the immunological responses seen in humans. For menopause studies, however, a further layer of sophistication is necessary. The mouse model must also support human ER signaling and simulate the gradual depletion of estrogen seen in perimenopausal and postmenopausal women.

Estrogen exerts its biological effects primarily through ERs, notably ERα, ERβ, and the G protein‐coupled estrogen receptor (GPER). These receptors are expressed in various target tissues, including the brain, bone, cardiovascular system, and immune cells, where they regulate gene transcription and mediate rapid non‐genomic signaling (Prossnitz and Barton 2009). During menopause, the downregulation of circulating estrogens results in altered ER expression and signaling, which contribute to tissue‐specific changes such as bone loss, endothelial dysfunction, and neuroinflammation (Ohlsson et al. 2012; Weitzmann and Pacifici 2006). Thus, for a humanized model to be truly reflective of menopausal physiology, it is essential to consider not just estrogen levels but also the dynamics of ER expression and function.

In addition to estrogen, the role of follicle‐stimulating hormone (FSH) must also be considered. FSH levels rise markedly in response to declining estrogen and have been increasingly implicated in postmenopausal pathophysiology, including bone loss and neurodegenerative diseases such as Alzheimer's disease (Jugulytė and Bartkevičienė 2025). Incorporating FSH signaling into a humanized mouse model could provide valuable insights into how elevated FSH levels interact with human immune and neural systems. Experimental approaches could include transgenic expression of human FSH receptors (FSHR) in relevant tissues or the administration of recombinant human FSH to mimic postmenopausal hormonal conditions, thereby allowing exploration of FSH‐immune and FSH‐brain interactions.

One approach to achieving this would be to create a dual‐humanized mouse model. In addition to hematopoietic reconstitution, the mouse would be engineered to express human ERs and potentially even human ovarian tissue. This could be accomplished by introducing human ovarian stromal cells or developing organoid‐based ovaries that mimic hormone secretion patterns observed in women. Alternatively, the OVX humanized mice could be supplemented with controlled‐release systems that mimic the declining estrogen curve characteristic of natural menopause. These hormone delivery systems could utilize bioengineered pellets or pumps to administer gradually decreasing doses of estradiol over a defined period, closely simulating the transition from pre‐ to post menopause. Moreover, the expression and distribution of ER subtypes can be evaluated in key organs during and after hormone depletion using immunohistochemical or transcriptomic approaches, allowing researchers to assess the impact of declining estrogen signaling on ER‐mediated pathways (Deroo and Korach 2006). This would provide critical insights into the selective role of ERα versus ERβ in tissue‐specific vulnerability and adaptation during menopause.

The integration of a human immune system in this model is especially critical for studying the intersection between estrogen signaling and immune responses. Menopause is associated with an increase in inflammatory markers and immune dysregulation, contributing to conditions such as osteoporosis, cardiovascular disease, and autoimmune disorders (McCarthy and Raval 2020). Humanized mice with immune systems derived from HSCs provide an opportunity to study how estrogen decline influences T cell aging, B cell repertoire, macrophage polarization, and cytokine production in a system that is far more analogous to human biology than murine models (Aryee et al. 2014). Furthermore, the inclusion of chronic low‐grade inflammation, or “inflammaging,” in model design would better reflect the immune environment of menopausal and aging women. This concept encompasses the persistent, systemic, low‐level inflammation that arises with age and contributes to diseases such as atherosclerosis, metabolic syndrome, and neurodegeneration. Assessing how declining estrogen levels exacerbate inflammaging and immune senescence, particularly the loss of naïve T cells, increased proinflammatory cytokine secretion, and altered macrophage function, would provide crucial insight into menopause‐associated immune aging.

ERs are also expressed in various immune cells, and their activation or suppression significantly influences immune homeostasis. For example, ERα signaling in T cells modulates pro‐inflammatory cytokine production, while ERβ activation in dendritic cells and macrophages can exert anti‐inflammatory effects (Kim et al. 2018). Importantly, estrogen exerts ambivalent and context‐dependent effects on immune regulation, which vary across life stages. High circulating estrogen levels during the reproductive years are associated with an increased risk of autoimmune diseases such as systemic lupus erythematosus (SLE), driven by enhanced B‐cell activation and antibody production. In contrast, lower estrogen levels after menopause are linked to a rise in inflammatory and degenerative conditions such as rheumatoid arthritis (RA) and osteoporosis. These opposing outcomes highlight that estrogen can act as either pro‐ or anti‐inflammatory depending on its concentration, receptor subtype engagement, and the immune cell type affected. Incorporating this complexity into humanized menopausal models will be crucial to accurately reflect how hormonal fluctuations shape immune responses throughout a woman's lifespan. Understanding how these ER‐mediated immune pathways shift during estrogen withdrawal will be essential in delineating menopause‐associated immune aging and vulnerability to inflammatory diseases.

It is also important to recognize that certain menopausal symptoms, such as hot flashes, are not directly replicable in mice due to species‐specific thermoregulatory mechanisms. However, mechanistic insights can be gained by studying thermoregulatory neurons and transient receptor potential (TRP) channels, such as TRPV1 and TRPM8, which are involved in heat and cold sensing. Investigating how estrogen and immune signaling influence TRP channel expression and activity in the hypothalamus could help approximate aspects of thermoregulation and vasomotor instability seen in menopause, even if overt hot flashes cannot be modeled.

However, there are several challenges inherent in developing and utilizing such a model. One major concern is the physiological compatibility between human immune cells and the murine environment. While humanized mice can replicate many aspects of human immune function, some components, such as the development of germinal centers or effective class‐switching of antibodies, are less robust in murine hosts (Alves da Costa et al. 2019; Chupp et al. 2024). Hormonal integration presents another layer of difficulty, as the interaction between human hormonal receptors and mouse tissue is not fully understood. Moreover, the generation of humanized mice is resource‐intensive, requiring specialized facilities, expertise in stem cell biology, and meticulous monitoring to ensure stable engraftment and reproducibility across cohorts.

Ethical considerations must also be considered. The use of human‐derived cells, particularly fetal tissue or stem cells, necessitates strict adherence to ethical guidelines and institutional review board (IRB) oversight. Animal welfare is another important concern, as the surgical procedures and immunodeficient states required for humanization can cause distress or increase susceptibility to infection. Therefore, experimental designs must incorporate the highest standards of care, including analgesics, barrier housing, and humane endpoints to minimize animal suffering.

Despite these challenges, the development of a humanized mouse model for menopause represents a groundbreaking step forward. It offers the potential to create a platform that can replicate the complexity of human menopause, including its hormonal, immune, skeletal, cardiovascular, and neurological dimensions. Such a model would not only advance our understanding of menopause itself but also serve as a preclinical testing ground for therapeutic interventions tailored to postmenopausal women. For instance, researchers could test the efficacy and safety of HRT, selective ER modulators (SERMs), or novel anti‐inflammatory agents in a context that closely mirrors human physiology.

One important consideration when designing OVX‐based humanized mouse models is the difference in reproductive cycle length between mice and humans. While mice exhibit a short estrous cycle of approximately 4–5 days, the human menstrual cycle lasts around 28 days, resulting in substantial differences in hormonal fluctuation patterns. Although OVX eliminates natural cycling, hormone replacement through pellets or osmotic pumps can be used to deliver estrogen and progesterone in a controlled, periodic manner to approximate human‐like hormonal rhythms. Incorporating cyclic hormone supplementation may improve the physiological relevance of these models, particularly for studying hormone‐immune or hormone‐bone interactions. However, full replication of the human menstrual cycle in mice remains biologically constrained due to species‐specific endocrine mechanisms. Therefore, adopting controlled, semi‐cyclic hormone delivery schedules in humanized mice represents a practical and translational compromise for simulating postmenopausal hormonal environments.

**FIGURE 4:**
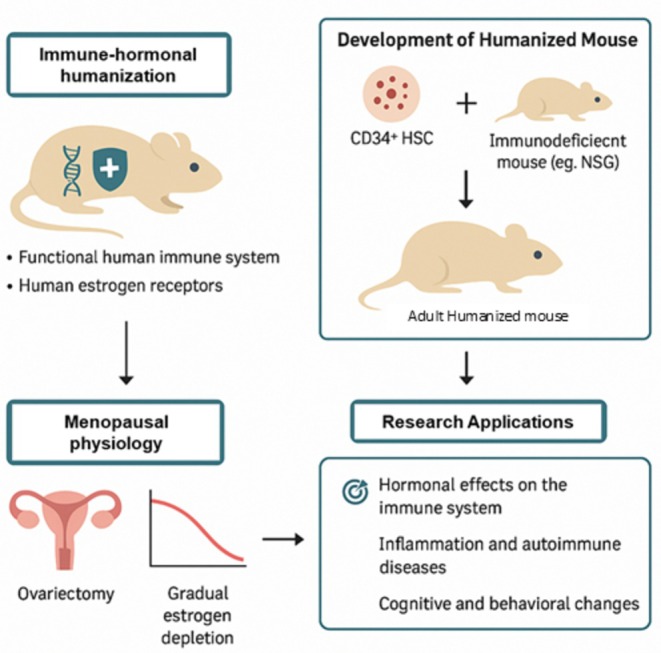
Schematic overview of proposed immune‐hormonal humanization in mice and its application in menopausal research. Humanized mice are generated by transplanting CD34^+^ human hematopoietic stem cells (HSCs) into immunodeficient mice, resulting in a functional human immune system and ERs. Ovariectomy is then used to induce abrupt estrogen depletion, modeling certain aspects of menopausal physiology. These models enable the study of hormonal influences on immunity, inflammation, autoimmune diseases, and cognitive or behavioral changes.

In conclusion, the proposed use of humanized mouse models in menopause research embodies a transformative strategy to address the gaps left by traditional animal models. By integrating human immune and hormonal components, researchers can delve deeper into the multifaceted nature of menopause, exploring both mechanistic pathways and therapeutic responses with unprecedented precision. While technical and ethical hurdles remain, the promise of these models in revolutionizing our approach to women's health during aging is undeniable. The next phase of menopause research could very well be defined by how successfully we adapt these advanced models to reflect the true human condition (Figure 4).

## 5 Functional Food and Menopause

**FIGURE 5:**
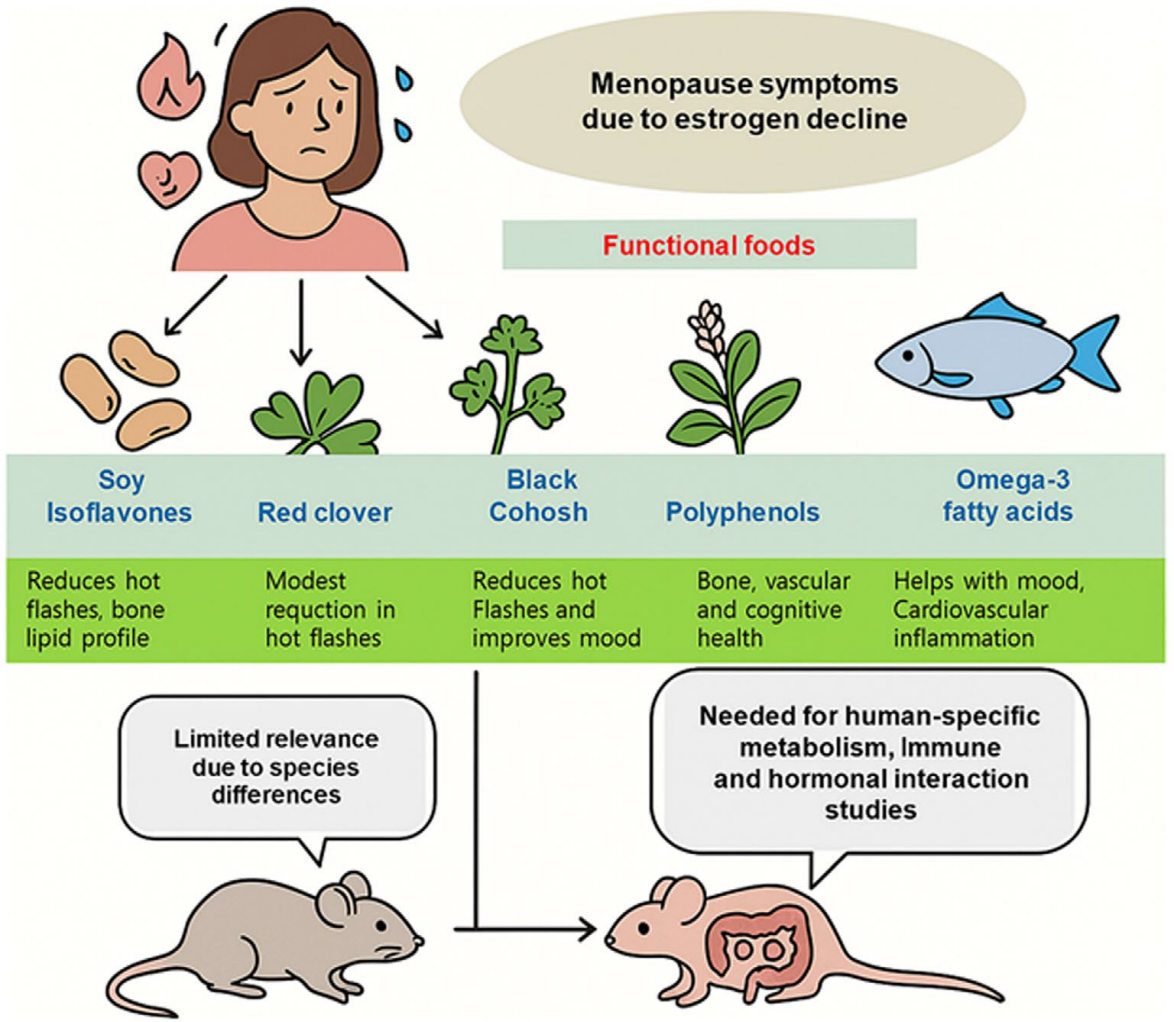
Overview of functional foods and their potential to alleviate menopause‐related symptoms, emphasizing the need for humanized mouse models in translational research. Menopause, driven by a decline in estrogen, leads to various symptoms such as hot flashes, mood changes, cardiovascular risk, and bone loss. Functional foods, such as soy isoflavones, red clover, black cohosh, polyphenols, and omega‐3 fatty acids, have demonstrated beneficial effects in mitigating these symptoms. While traditional rodent models (left) have been widely used to evaluate these effects, their limited translational relevance due to species‐specific differences in metabolism, immunity, and hormonal regulation poses challenges. Humanized mouse models (right), incorporating human immune systems, hormonal profiles, or microbiota, offer a promising platform for assessing the efficacy, safety, and mechanisms of these compounds in a physiologically relevant context.

Functional foods have emerged as a promising approach to mitigating the adverse effects associated with menopause (De Franciscis et al. 2019) (Figure 5). These foods, rich in bioactive compounds, have demonstrated various health benefits in both preclinical and clinical settings, particularly in the context of managing symptoms related to estrogen decline, such as osteoporosis, cardiovascular risk, hot flashes, and mood disturbances (Meegaswatte et al. 2024; Zukić et al. 2024). Despite a growing body of literature supporting the use of functional foods for menopausal symptom relief, a significant limitation persists: the absence of robust studies using humanized mouse models to explore these interactions in a physiologically relevant context.

Among the most extensively studied functional food components in menopause research are soy isoflavones. These phytoestrogens, predominantly genistein and daidzein, mimic the effects of endogenous estrogen by binding to ERs, particularly ER‐β, with high affinity (Pejčić et al. 2023). Numerous clinical trials have reported improvements in vasomotor symptoms, bone mineral density, and lipid profiles in postmenopausal women who consume soy‐based diets or isoflavone supplements (Lee et al. 2017). Preclinical studies in rodent models, especially OVX mice and rats, have also shown favorable outcomes, including protection against bone loss and modulation of inflammatory cytokines (Jayusman et al. 2023). However, these models lack the human immune and hormonal milieu, which could significantly influence isoflavone metabolism and efficacy.

Red clover, another plant source rich in isoflavones, has similarly been investigated for its potential to alleviate menopausal symptoms. Clinical studies have yielded mixed results, with some showing modest reductions in hot flashes and others reporting no significant benefit. Its effectiveness is thought to depend on the presence of gut microbiota capable of converting isoflavones into bioactive forms such as equol (Burdette et al. 2002; Shakeri et al. 2015). Again, this highlights the complexity of human‐specific metabolic pathways, which cannot be adequately replicated in standard rodent models. The use of humanized mice with transplanted human gut microbiota and immune systems could provide deeper insights into how red clover and similar compounds exert their effects in a more relevant physiological environment.

Black cohosh, a North American herb widely used as an alternative therapy for menopausal symptoms, has shown efficacy in reducing hot flashes and improving mood, although its mechanism of action remains unclear (Leach and Moore 2012). Unlike isoflavones, black cohosh does not appear to bind directly to ERs, suggesting that its effects may be mediated through serotonergic or inflammatory pathways (Ruhlen et al. 2008). While there is substantial anecdotal and clinical evidence supporting its use, preclinical studies remain limited, and none to date have explored its effects in humanized mouse models that could provide clarity on its pharmacodynamics and long‐term safety.

Polyphenols, including flavonoids found in berries, tea, and cocoa, have garnered attention for their antioxidant and anti‐inflammatory properties. In the context of menopause, polyphenols may contribute to the preservation of bone health, vascular function, and cognitive performance (Liu et al. 2024). Clinical data have indicated potential benefits in lipid metabolism and endothelial function among postmenopausal women consuming polyphenol‐rich diets (Sánchez‐Martínez et al. 2021). Animal studies reinforce these findings, but, as with other compounds, they fail to account for the complexity of human‐specific metabolism, immune responses, and hormonal interactions. Humanized mouse models could bridge this gap by allowing researchers to observe how polyphenols interact with human immune cells, microbiota, and estrogen pathways in a controlled experimental setting.

Omega‐3 fatty acids, primarily derived from fish oil, have shown promise in managing menopausal symptoms, particularly those related to mood, cardiovascular health, and inflammation (Raza et al. 2025). Studies have demonstrated that omega‐3 supplementation may reduce depressive symptoms and improve heart rate variability and lipid profiles in postmenopausal women (Persons et al. 2014). Animal studies support its anti‐inflammatory effects and benefits for brain and cardiovascular health (Zivkovic et al. 2011). However, the translation of these findings to humans remains partially limited by differences in lipid metabolism and immune function across species. The use of humanized mice could allow for a more accurate assessment of how omega‐3s influence immune‐inflammatory signaling in a menopausal context, offering valuable insights into personalized nutritional therapies.

Probiotics represent another emerging frontier in menopause research. The gut microbiome is increasingly recognized for its role in modulating systemic inflammation, estrogen metabolism, and immune responses (Peters et al. 2022). Some studies suggest that probiotic supplementation can improve vaginal health, metabolic parameters, and mood in postmenopausal women. In animal models, probiotics have demonstrated protective effects against bone loss and systemic inflammation (Lee et al. 2021; Yu et al. 2024), yet the human relevance of these results is constrained by interspecies differences in microbiota composition and immune‐epithelial crosstalk. Humanized mice colonized with human microbiota offer an exciting avenue for investigating how probiotics might influence the estrogen‐microbiome‐immune axis in menopausal physiology.

Despite this growing landscape of research on functional foods and menopause, a striking gap exists in the integration of humanized mouse models into these studies. Most preclinical research continues to rely on traditional rodent models that, while useful, do not fully replicate the human hormonal, immune, or metabolic environments. The absence of humanized models limits our ability to understand how these bioactive compounds truly function within human‐like systems. This is particularly problematic for compounds whose bioactivity is influenced by human‐specific pathways, such as ER subtypes, liver metabolism, or gut microbial processing. Moreover, even when humanized mice are used, several experimental challenges remain. The metabolism of nutrients and bioactive compounds may still differ between humans and hu‐mice, leading to discrepancies in absorption, distribution, and clearance. Determining nutritionally relevant dosing regimens that accurately reflect human dietary intake is complex and requires careful calibration. Without proper pharmacokinetic validation, there is a risk of overestimating or underestimating the true physiological impact of functional food compounds. Therefore, future studies should include dose translation strategies, such as body surface area–based scaling, and employ biomarker or metabolite profiling to confirm human‐relevant exposure levels. These considerations are essential for ensuring the reliability and translational value of nutritional interventions in hu‐mice models.

Given the increasing interest in personalized medicine and nutrition, there is a critical need to develop and utilize humanized mouse models for testing functional foods in the context of menopause. Such models would allow researchers to assess not only the efficacy of these compounds but also their safety, metabolism, and potential for interaction with other physiological systems. For instance, a humanized mouse with both immune and hormonal humanization could be used to test the impact of soy isoflavones or polyphenols on bone density, immune aging, and cardiovascular health during and after induced menopause. These studies could also provide valuable data on interindividual differences in response to dietary interventions, paving the way for more targeted nutritional strategies.

In conclusion, functional foods offer a promising complementary approach to managing menopausal symptoms, with various compounds showing benefits in clinical and animal studies. However, the full translational potential of these findings remains untapped due to the lack of advanced preclinical models that accurately reflect human biology. Integrating appropriate dosing validation, metabolic profiling, and human‐specific calibration into hu‐mice studies will be pivotal for maximizing translational accuracy and minimizing interspecies discrepancies. The integration of humanized mouse models into this field represents a transformative step that could bridge the existing gap between bench and bedside, ultimately enhancing our ability to design effective, safe, and personalized dietary interventions for women undergoing menopause.

## 6 Future Perspectives: Bridging Traditional Menopause Research With Modern Immune‐Based Models

Future perspectives in menopause research necessitate a paradigm shift from traditional models toward more sophisticated and human‐relevant systems that can unravel the complex interplay between hormonal, immune, and metabolic pathways. Menopause, long studied through OVX rodent models, has often been explored with a primary focus on hormonal depletion and its direct physiological outcomes such as bone loss or hot flashes. While these studies have provided foundational knowledge, they fall short in accounting for the broader systemic effects seen in women, particularly those modulated by the human immune system, microbiota, and individualized metabolic responses. Bridging this gap requires not only technological innovation but also an integrated, interdisciplinary research strategy. Humanized mouse models, engineered to harbor human immune cells, and ideally human hormonal and metabolic components, offer a promising frontier to realize this goal.

One of the most compelling opportunities lies in combining the use of humanized mice with functional food studies to explore immune‐based effects in menopause. As discussed in previous sections, functional foods such as soy isoflavones, omega‐3 fatty acids, and probiotics have shown beneficial effects in alleviating menopausal symptoms through pathways that likely involve immune and metabolic regulation. However, these effects are highly dependent on human‐specific mechanisms including ER signaling, cytokine interactions, and the gut microbiome. Standard rodent models simply do not capture the nuance of these pathways, which limits their utility in translating findings to human populations. Humanized mice, particularly those reconstituted with human immune systems and potentially colonized with human microbiota, provide an unprecedented opportunity to test how these functional foods interact with human‐like immune‐hormonal networks under menopausal conditions.

Using humanized models can also help validate emerging hypotheses about the immunological basis of menopause‐related conditions. For example, chronic low‐grade inflammation has been identified as a key driver of postmenopausal pathologies such as osteoporosis, cardiovascular disease, and neurodegeneration. With humanized mice, researchers can observe how menopause‐induced hormonal changes influence human immune cells, such as T‐regulatory cells, macrophages, or dendritic cells, in real time. Furthermore, the impact of functional foods on these immune pathways can be directly assessed, allowing for precise delineation of mechanisms and identification of bioactive compounds with true immunomodulatory potential in human physiology.

An additional challenge in current menopausal research is the frequent use of young mice in experimental settings, which does not accurately represent the physiological context of postmenopausal women who are typically older and experience age‐related complications such as osteoporosis, metabolic decline, and chronic inflammation. Age profoundly influences immune function, bone metabolism, and hormonal responsiveness; thus, using young rodents can underestimate or misrepresent the true postmenopausal phenotype. To address this, future studies should consider employing aged humanized mice, or integrating human immune and stromal cells derived from older donors, to more closely mimic the biological state of postmenopausal women. This approach would enable the investigation of how aging synergistically interacts with hormonal loss to drive pathologies like osteoporosis and frailty, providing a more accurate and translational model for therapeutic discovery.

Beyond mechanistic insights, humanized models can also serve as valuable platforms for testing personalized nutritional interventions. Women experience menopause in highly individualized ways, influenced by genetic, epigenetic, dietary, and environmental factors. By using human cells derived from diverse donors, it may be possible to create stratified humanized models that mimic this variability, offering a testbed for personalized approaches in menopause therapy. For instance, researchers could investigate whether a particular isoflavone‐rich diet produces different effects in humanized mice derived from donors with distinct ER genotypes or immune profiles. This would allow for a better understanding of who might benefit most from specific dietary interventions and why, ultimately supporting the move toward precision nutrition.

The successful realization of this integrative model requires collaborative efforts across disciplines. Endocrinologists, immunologists, nutrition scientists, and biomedical engineers must come together to design models that not only simulate human menopause more accurately but also account for the multifactorial nature of aging in women. Collaboration with computational modelers and bioinformaticians could further enhance this framework by enabling systems‐level analysis of large datasets generated from these studies, ranging from transcriptomic and metabolomic profiles to immune cell phenotyping and microbiome composition. These collaborations can drive the development of holistic models of menopause that incorporate immune, hormonal, metabolic, and dietary dimensions.

Furthermore, ethical considerations and regulatory standards must evolve in tandem with these scientific advancements. Humanized mice, particularly those developed from stem cells or fetal tissues, raise important ethical questions regarding donor consent, tissue sourcing, and long‐term use. Transparent and standardized protocols, along with clear ethical oversight, are essential to ensure that this research progresses responsibly. Regulatory bodies should also work with scientists to develop guidelines for using data from humanized models in the approval of functional foods or supplements aimed at menopausal women, bridging preclinical research with human health applications.

In the context of global health, integrating humanized models into menopause research is especially relevant as the global population ages and the demand for effective, safe, and accessible menopausal therapies continues to grow. Functional foods represent a relatively low‐cost, culturally adaptable, and non‐pharmaceutical strategy that can be deployed in diverse settings. Validating their effects in models that more accurately mimic human biology not only strengthens the scientific basis for their use but also accelerates their adoption in public health strategies worldwide.

**FIGURE 6:**
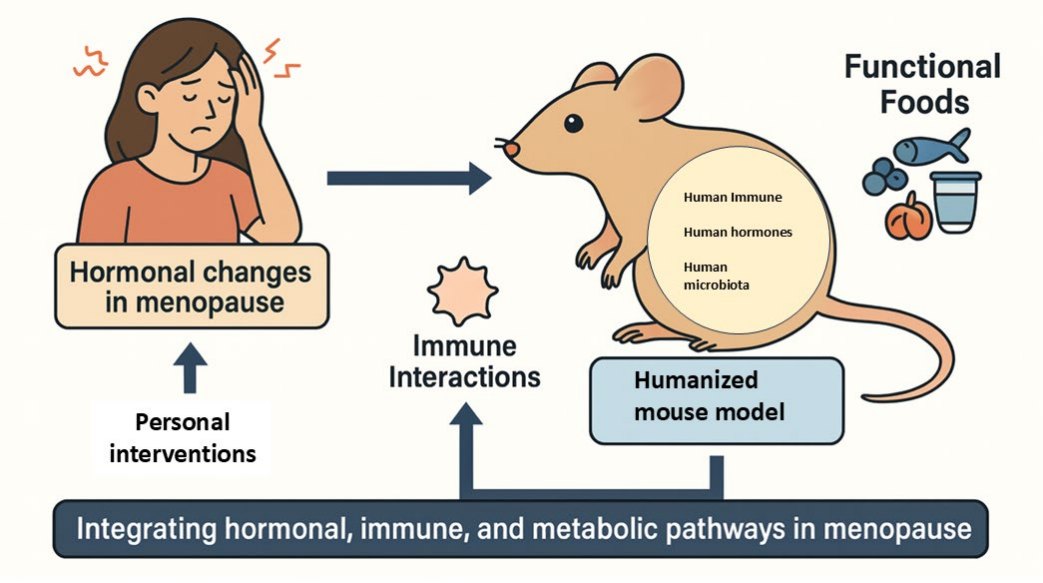
Future perspectives in menopause research: Bridging traditional models with humanized immune‐based platforms. Evolution of menopause research from traditional ovariectomized (OVX) rodent models focused mainly on hormonal depletion toward advanced humanized mouse models that integrate human immune cells, microbiota, and metabolic profiles. It highlights how these humanized systems can better capture the complex interactions between hormones, immune responses, and metabolism, offering deeper insights into menopause‐related pathologies. The incorporation of functional food interventions is depicted, showing their potential to modulate immune and metabolic pathways in a human‐relevant context.

In summary, future directions in menopause research must prioritize the incorporation of humanized mouse models to overcome the limitations of traditional animal models and to better capture the intricate interplay between hormones, immunity, and diet (Figure 6). This shift offers the potential for deeper mechanistic understanding, more accurate predictive models, and ultimately, the development of tailored interventions that can improve the quality of life for women globally. The integration of functional food research into this platform opens a new horizon in personalized medicine, one where nutrition and biology converge to offer meaningful, evidence‐based solutions for menopause management.

## 7 Conclusion

Menopause research stands to benefit greatly from the integration of humanized mouse models, which offer a more accurate representation of human immune and hormonal systems than traditional models. These advanced tools can help uncover the complex mechanisms driving menopausal symptoms and related diseases. Additionally, functional foods show promise as safe, non‐invasive interventions, but their effects need further validation in human‐relevant models. By combining these two approaches, researchers can develop more effective, personalized strategies to support women's health during and after menopause.

## Author Contributions

**Nisansala Chandimali**, **Jaehoon Bae**, and **Sun Hee Cheong:** investigation, writing – original draft. **Seon Gyeong Bak** and **Yeon‐Yong Kim:** validation, funding acquisition. **Ji‐Su Kim** and **Seung‐Jae Lee:** conceptualization, project administration, funding acquisition, supervision, writing – review and editing.

## Funding

This work was supported by several grants: the KRIBB Research Initiative Program (KGM1052511 and 5162524), the Basic Science Research Program through the NRF, funded by the Ministry of Education, Science, and Technology (NRF‐2022R1A6A3A01087055 and NRF‐2021R1C1C2095006). This research was supported by the Regional Innovation System and Education (RISE) program through the Gyeongbuk and Jeonbuk RISE Center, funded by the Ministry of Education (MOE) and the Gyeongsangbuk and Jeonbuk State, Republic of Korea (2025‐RISE‐13‐WSU).

## Conflicts of Interest

The authors declare no conflicts of interest.

## Data Availability

The authors have nothing to report.

## References

[acel70313-bib-0001] Alves da Costa, T. , J. Lang , R. M. Torres , and R. Pelanda . 2019. “The Development of Human Immune System Mice and Their Use to Study Tolerance and Autoimmunity.” Journal of Translational Autoimmunity 2: 100021. 10.1016/j.jtauto.2019.100021. 32743507 PMC7388352

[acel70313-bib-0002] Aryee, K. E. , L. D. Shultz , and M. A. Brehm . 2014. “Immunodeficient Mouse Model for Human Hematopoietic Stem Cell Engraftment and Immune System Development.” Methods in Molecular Biology 1185: 267–278. 10.1007/978-1-4939-1133-2_18. 25062635 PMC4476265

[acel70313-bib-0003] Baeza, I. , N. M. De Castro , L. Giménez‐Llort , and M. De la Fuente . 2010. “Ovariectomy, a Model of Menopause in Rodents, Causes a Premature Aging of the Nervous and Immune Systems.” Journal of Neuroimmunology 219, no. 1‐2: 90–99. 10.1016/j.jneuroim.2009.12.008. 20096467

[acel70313-bib-0004] Balestri, W. , R. Sharma , V. A. da Silva , et al. 2024. “Modeling the Neuroimmune System in Alzheimer's and Parkinson's Diseases.” Journal of Neuroinflammation 21, no. 1: 32. 10.1186/s12974-024-03024-8. 38263227 PMC10807115

[acel70313-bib-0005] Brehm, M. A. , M. V. Wiles , D. L. Greiner , and L. D. Shultz . 2014. “Generation of Improved Humanized Mouse Models for Human Infectious Diseases.” Journal of Immunological Methods 410: 3–17. 10.1016/j.jim.2014.02.011. 24607601 PMC4155027

[acel70313-bib-0006] Burdette, J. E. , J. Liu , D. Lantvit , et al. 2002. “ *Trifolium pratense* (Red Clover) Exhibits Estrogenic Effects In Vivo in Ovariectomized Sprague‐Dawley Rats.” Journal of Nutrition 132, no. 1: 27–30. 10.1093/jn/132.1.27. 11773503

[acel70313-bib-0007] Camon, C. , M. Garratt , and S. M. Correa . 2024. “Exploring the Effects of Estrogen Deficiency and Aging on Organismal Homeostasis During Menopause.” Nature Aging 4, no. 12: 1731–1744. 10.1038/s43587-024-00767-0. 39672893 PMC11785355

[acel70313-bib-0008] Chalvon‐Demersay, T. , F. Blachier , D. Tomé , and A. Blais . 2017. “Animal Models for the Study of the Relationships Between Diet and Obesity: A Focus on Dietary Protein and Estrogen Deficiency.” Frontiers in Nutrition 4: 5. 10.3389/fnut.2017.00005. 28373974 PMC5357654

[acel70313-bib-0009] Chen, A. , I. Neuwirth , and D. Herndler‐Brandstetter . 2023. “Modeling the Tumor Microenvironment and Cancer Immunotherapy in Next‐Generation Humanized Mice.” Cancers (Basel) 15, no. 11: 2989. 10.3390/cancers15112989. 37296949 PMC10251926

[acel70313-bib-0010] Chen, J. , S. Liao , H. Zhou , et al. 2022. “Humanized Mouse Models of Systemic Lupus Erythematosus: Opportunities and Challenges.” Frontiers in Immunology 12: 816956. 10.3389/fimmu.2021.816956. 35116040 PMC8804209

[acel70313-bib-0011] Chen, P. , B. Li , and L. Ou‐Yang . 2022. “Role of Estrogen Receptors in Health and Disease.” Front Endocrinol (Lausanne) 13: 839005. 10.3389/fendo.2022.839005. 36060947 PMC9433670

[acel70313-bib-0012] Chupp, D. P. , C. E. Rivera , Y. Zhou , et al. 2024. “A Humanized Mouse That Mounts Mature Class‐Switched, Hypermutated and Neutralizing Antibody Responses.” Nature Immunology 25, no. 8: 1489–1506. 10.1038/s41590-024-01880-3. 38918608 PMC11291283

[acel70313-bib-0013] Chuprin, J. , H. Buettner , M. O. Seedhom , et al. 2023. “Humanized Mouse Models for Immuno‐Oncology Research.” Nature Reviews Clinical Oncology 20, no. 3: 192–206. 10.1038/s41571-022-00721-2. PMC10593256 36635480

[acel70313-bib-0014] Dash, P. K. , S. Gorantla , L. Poluektova , et al. 2021. “Humanized Mice for Infectious and Neurodegenerative Disorders.” Retrovirology 18, no. 1: 13. 10.1186/s12977-021-00557-1. 34090462 PMC8179712

[acel70313-bib-0015] De Franciscis, P. , N. Colacurci , G. Riemma , et al. 2019. “A Nutraceutical Approach to Menopausal Complaints.” Medicina (Kaunas, Lithuania) 55, no. 9: 544. 10.3390/medicina55090544. 31466381 PMC6780855

[acel70313-bib-0016] Deroo, B. J. , and K. S. Korach . 2006. “Estrogen Receptors and Human Disease.” Journal of Clinical Investigation 116, no. 3: 561–570. 10.1172/JCI27987. 16511588 PMC2373424

[acel70313-bib-0017] Diaz Brinton, R. 2012. “Minireview: Translational Animal Models of Human Menopause: Challenges and Emerging Opportunities.” Endocrinology 153, no. 8: 3571–3578. 10.1210/en.2012-1340. 22778227 PMC3404353

[acel70313-bib-0018] Dong, S. , C. Fu , C. Shu , et al. 2024. “Development of a Humanized Mouse Model With Functional Human Materno‐Fetal Interface Immunity.” JCI Insight 9, no. 20: e176527. 10.1172/jci.insight.176527. 39435662 PMC11529984

[acel70313-bib-0019] Freedman, R. R. 2014. “Menopausal Hot Flashes: Mechanisms, Endocrinology, Treatment.” Journal of Steroid Biochemistry and Molecular Biology 142: 115–120. 10.1016/j.jsbmb.2013.08.010. 24012626 PMC4612529

[acel70313-bib-0020] Fu, S. , J. Wang , W. Sun , Y. Xu , X. Zhou , and W. Cheng . 2014. “Preclinical Humanized Mouse Model With Ectopic Ovarian Tissues.” Experimental and Therapeutic Medicine 8, no. 3: 742–746. 10.3892/etm.2014.1819. 25120592 PMC4113642

[acel70313-bib-0021] He, Y. , B. Liang , S. W. Hung , et al. 2022. “Re‐Evaluation of Mouse Models of Endometriosis for Pathological and Immunological Research.” Frontiers in Immunology 13: 986202. 10.3389/fimmu.2022.986202. 36466829 PMC9716019

[acel70313-bib-0022] Jayusman, P. A. , N. S. Nasruddin , B. Baharin , N. Ibrahim , H. Ahmad Hairi , and A. N. Shuid . 2023. “Overview on Postmenopausal Osteoporosis and Periodontitis: The Therapeutic Potential of Phytoestrogens Against Alveolar Bone Loss.” Frontiers in Pharmacology 14: 1120457. 10.3389/fphar.2023.1120457. 36909165 PMC9995413

[acel70313-bib-0023] Jiang, R. D. , M. Q. Liu , Y. Chen , et al. 2020. “Pathogenesis of SARS‐CoV‐2 in Transgenic Mice Expressing Human Angiotensin‐Converting Enzyme 2.” Cell 182, no. 1: 50–58. 10.1016/j.cell.2020.05.027. 32516571 PMC7241398

[acel70313-bib-0072] Jugulytė, N., and D. Bartkevičienė . 2025. “The Role of Follicle‐Stimulating Hormone in Bone Loss During Menopause Transition: A Narrative Review.” Endocrines 6, no. 4: 54. 10.3390/endocrines6040054..

[acel70313-bib-0024] Kan, L. 2013. “Chapter 16‐Animal Models of Bone Diseases‐A.” In Animal Models for the Study of Human Disease, edited by P. M. Conn , 353–390. Academic Press.

[acel70313-bib-0025] Kim, R. Y. , D. Mangu , A. S. Hoffman , et al. 2018. “Oestrogen Receptor β Ligand Acts on CD11c^+^ Cells to Mediate Protection in Experimental Autoimmune Encephalomyelitis.” Brain 141, no. 1: 132–147. 10.1093/brain/awx315. 29228214 PMC5837360

[acel70313-bib-0026] Kisoh, K. , G. Sugahara , Y. Ogawa , et al. 2021. “Estimating Drug Efficacy With a Diet‐Induced NASH Model in Chimeric Mice With Humanized Livers.” Biomedicine 9, no. 11: 1647. 10.3390/biomedicines9111647. PMC8615377 34829876

[acel70313-bib-0027] Koh, N. Y. Y. , J. J. Miszkiewicz , M. L. Fac , N. K. Y. Wee , and N. A. Sims . 2024. “Preclinical Rodent Models for Human Bone Disease, Including a Focus on Cortical Bone.” Endocrine Reviews 45, no. 4: 493–520. 10.1210/endrev/bnae004. 38315213 PMC11244217

[acel70313-bib-0028] Kozieł, M. J. , and A. W. Piastowska‐Ciesielska . 2023. “Estrogens, Estrogen Receptors and Tumor Microenvironment in Ovarian Cancer.” International Journal of Molecular Sciences 24, no. 19: 14673. 10.3390/ijms241914673. 37834120 PMC10572993

[acel70313-bib-0029] Lai, F. , and Q. Chen . 2018. “Humanized Mouse Models for the Study of Infection and Pathogenesis of Human Viruses.” Viruses 10, no. 11: 643. 10.3390/v10110643. 30453598 PMC6266013

[acel70313-bib-0030] Leach, M. J. , and V. Moore . 2012. “Black Cohosh (Cimicifuga spp.) for Menopausal Symptoms.” Cochrane Database of Systematic Reviews 2012, no. 9: Cd007244. 10.1002/14651858.CD007244.pub2. 22972105 PMC6599854

[acel70313-bib-0031] Lee, H. , R. Choue , and H. Lim . 2017. “Effect of Soy Isoflavones Supplement on Climacteric Symptoms, Bone Biomarkers, and Quality of Life in Korean Postmenopausal Women: A Randomized Clinical Trial.” Nutrition Research and Practice 11, no. 3: 223–231. 10.4162/nrp.2017.11.3.223. 28584579 PMC5449379

[acel70313-bib-0032] Lee, S. , D. H. Jung , M. Park , et al. 2021. “The Effect of *Lactobacillus gasseri* BNR17 on Postmenopausal Symptoms in Ovariectomized Rats.” Journal of Microbiology and Biotechnology 31, no. 9: 1281–1287. 10.4014/jmb.2105.05032. 34319260 PMC9705893

[acel70313-bib-0033] Liu, M. q. , and X. Yang . 2025. “Patient‐Derived Xenograft Models: Current Status, Challenges, and Innovations in Cancer Research.” Genes and Diseases 12: 101520. 10.1016/j.gendis.2025.101520. 40548062 PMC12179623

[acel70313-bib-0034] Liu, Y. , M. Fang , X. Tu , et al. 2024. “Dietary Polyphenols as Anti‐Aging Agents: Targeting the Hallmarks of Aging.” Nutrients 16, no. 19: 3305. 10.3390/nu16193305. 39408272 PMC11478989

[acel70313-bib-0035] Liu, Y. , W. Wu , C. Cai , H. Zhang , H. Shen , and Y. Han . 2023. “Patient‐Derived Xenograft Models in Cancer Therapy: Technologies and Applications.” Signal Transduction and Targeted Therapy 8, no. 1: 160. 10.1038/s41392-023-01419-2. 37045827 PMC10097874

[acel70313-bib-0036] Lõhmussaar, K. , M. Boretto , and H. Clevers . 2020. “Human‐Derived Model Systems in Gynecological Cancer Research.” Trends in Cancer 6, no. 12: 1031–1043. 10.1016/j.trecan.2020.07.007. 32855097

[acel70313-bib-0037] Malvezzi, H. , E. B. Marengo , S. Podgaec , and C. A. Piccinato . 2020. “Endometriosis: Current Challenges in Modeling a Multifactorial Disease of Unknown Etiology.” Journal of Translational Medicine 18, no. 1: 311. 10.1186/s12967-020-02471-0. 32787880 PMC7425005

[acel70313-bib-0038] McCarthy, M. , and A. P. Raval . 2020. “The Peri‐Menopause in a Woman's Life: A Systemic Inflammatory Phase That Enables Later Neurodegenerative Disease.” Journal of Neuroinflammation 17, no. 1: 317. 10.1186/s12974-020-01998-9. 33097048 PMC7585188

[acel70313-bib-0039] Medina‐Contreras, J. , R. Villalobos‐Molina , A. Zarain‐Herzberg , and J. Balderas‐Villalobos . 2020. “Ovariectomized Rodents as a Menopausal Metabolic Syndrome Model. A Minireview.” Molecular and Cellular Biochemistry 475, no. 1–2: 261–276. 10.1007/s11010-020-03879-4. 32852713

[acel70313-bib-0040] Meegaswatte, H. , K. Speer , A. J. McKune , and N. Naumovski . 2024. “Functional Foods and Nutraceuticals for the Management of Cardiovascular Disease Risk in Postmenopausal Women.” Reviews in Cardiovascular Medicine 25, no. 12: 460. 10.31083/j.rcm2512460. 39742223 PMC11683719

[acel70313-bib-0041] Méndez, L. , and I. Medina . 2021. “Polyphenols and Fish Oils for Improving Metabolic Health: A Revision of the Recent Evidence for Their Combined Nutraceutical Effects.” Molecules 26, no. 9: 2438. 10.3390/molecules26092438. 33922113 PMC8122614

[acel70313-bib-0042] Negi, S. , S. Saini , N. Tandel , K. Sahu , R. P. N. Mishra , and R. K. Tyagi . 2021. “Translating Treg Therapy for Inflammatory Bowel Disease in Humanized Mice.” Cells 10, no. 8: 1847. 10.3390/cells10081847. 34440615 PMC8393385

[acel70313-bib-0043] Nieto, M. R. , M. J. Rus , V. Areal‐Quecuty , D. M. Lubián‐López , and A. Simon‐Soro . 2025. “Menopausal Shift on Women's Health and Microbial Niches.” Npj Women's Health 3, no. 1: 3. 10.1038/s44294-024-00050-y.

[acel70313-bib-0044] Ohlsson, C. , C. Engdahl , A. E. Börjesson , et al. 2012. “Estrogen Receptor‐α Expression in Neuronal Cells Affects Bone Mass.” Proceedings of the National Academy of Sciences of the United States of America 109, no. 3: 983–988. 10.1073/pnas.1111436109. 22215598 PMC3271867

[acel70313-bib-0045] Park, C.‐K. , M. Khalil , N.‐A. Pham , et al. 2025. “Humanized Mouse Models for Immuno‐Oncology Research: A Review and Implications in Lung Cancer Research.” JTO Clinical and Research Reports 6, no. 3: 100781. 10.1016/j.jtocrr.2024.100781. 39990135 PMC11847118

[acel70313-bib-0046] Pejčić, T. , M. Zeković , U. Bumbaširević , et al. 2023. “The Role of Isoflavones in the Prevention of Breast Cancer and Prostate Cancer.” Antioxidants (Basel) 12, no. 2: 368. 10.3390/antiox12020368. 36829927 PMC9952119

[acel70313-bib-0047] Persons, J. E. , J. G. Robinson , E. M. Ammann , et al. 2014. “Omega‐3 Fatty Acid Biomarkers and Subsequent Depressive Symptoms.” International Journal of Geriatric Psychiatry 29, no. 7: 747–757. 10.1002/gps.4058. 24338726 PMC4048630

[acel70313-bib-0048] Peters, B. A. , N. Santoro , R. C. Kaplan , and Q. Qi . 2022. “Spotlight on the Gut Microbiome in Menopause: Current Insights.” International Journal of Women's Health 14: 1059–1072. 10.2147/ijwh.S340491. PMC9379122 35983178

[acel70313-bib-0049] Prossnitz, E. R. , and M. Barton . 2009. “Signaling, Physiological Functions and Clinical Relevance of the G Protein‐Coupled Estrogen Receptor GPER.” Prostaglandins & Other Lipid Mediators 89, no. 3–4: 89–97. 10.1016/j.prostaglandins.2009.05.001. 19442754 PMC2740807

[acel70313-bib-0050] Raza, M. L. , S. T. Hassan , S. Jamil , W. Fatima , and M. Fatima . 2025. “Nutritional Interventions in Depression: The Role of Vitamin D and Omega‐3 Fatty Acids in Neuropsychiatric Health.” Clinical Nutrition 45: 270–280. 10.1016/j.clnu.2025.01.009. 39874718

[acel70313-bib-0052] Ruhlen, R. L. , G. Y. Sun , and E. R. Sauter . 2008. “Black Cohosh: Insights Into Its Mechanism(s) of Action.” Integr Med Insights 3: 21–32. 21614156 PMC3046019

[acel70313-bib-0053] Sadeghi, M. R. 2022. “Polycystic Ovarian Syndrome and Endometriosis as Two Evil Extremes of Health Continuum.” Journal of Reproductive and Infertility 23, no. 1: 1–2. 10.18502/jri.v23i1.8445. PMC9361725 36045878

[acel70313-bib-0054] Samuelson, C. , S. Radtke , H. Zhu , et al. 2021. “Multiplex CRISPR/Cas9 Genome Editing in Hematopoietic Stem Cells for Fetal Hemoglobin Reinduction Generates Chromosomal Translocations.” Molecular Therapy 23: 507–523. 10.1016/j.omtm.2021.10.008. 34853798 PMC8605315

[acel70313-bib-0055] Sánchez‐Martínez, L. , M. J. Periago , J. García‐Alonso , M. T. García‐Conesa , and R. González‐Barrio . 2021. “A Systematic Review of the Cardiometabolic Benefits of Plant Products Containing Mixed Phenolics and Polyphenols in Postmenopausal Women: Insufficient Evidence for Recommendations to This Specific Population.” Nutrients 13, no. 12: 4276. 10.3390/nu13124276. 34959828 PMC8707028

[acel70313-bib-0056] Saxena, R. , M. Nassiri , X. M. Yin , and N. Morral . 2022. “Insights From a High‐Fat Diet Fed Mouse Model With a Humanized Liver.” PLoS One 17, no. 5: e0268260. 10.1371/journal.pone.0268260. 35533183 PMC9084523

[acel70313-bib-0057] Scherer, S. D. , A. I. Riggio , F. Haroun , et al. 2021. “An Immune‐Humanized Patient‐Derived Xenograft Model of Estrogen‐Independent, Hormone Receptor Positive Metastatic Breast Cancer.” Breast Cancer Research 23, no. 1: 100. 10.1186/s13058-021-01476-x. 34717714 PMC8556932

[acel70313-bib-0058] Schinnerling, K. , C. Rosas , L. Soto , R. Thomas , and J. C. Aguillón . 2019. “Humanized Mouse Models of Rheumatoid Arthritis for Studies on Immunopathogenesis and Preclinical Testing of Cell‐Based Therapies.” Frontiers in Immunology 10: 203. 10.3389/fimmu.2019.00203. 30837986 PMC6389733

[acel70313-bib-0059] Shakeri, F. , S. Taavoni , A. Goushegir , and H. Haghani . 2015. “Effectiveness of Red Clover in Alleviating Menopausal Symptoms: A 12‐Week Randomized, Controlled Trial.” Climacteric: The Journal of the International Menopause Society 18: 1–17. 10.3109/13697137.2014.999660. 25581426

[acel70313-bib-0060] Shultz, L. D. , M. A. Brehm , J. V. Garcia‐Martinez , and D. L. Greiner . 2012. “Humanized Mice for Immune System Investigation: Progress, Promise and Challenges.” Nature Reviews. Immunology 12, no. 11: 786–798. 10.1038/nri3311. PMC3749872 23059428

[acel70313-bib-0061] Tello, J. A. , H. E. Williams , R. M. Eppler , M. L. Steinhilb , and M. Khanna . 2022. “Animal Models of Neurodegenerative Disease: Recent Advances in Fly Highlight Innovative Approaches to Drug Discovery.” Frontiers in Molecular Neuroscience 15: 883358. 10.3389/fnmol.2022.883358. 35514431 PMC9063566

[acel70313-bib-0062] Tyagi, R. K. , J. Li , J. Jacobse , S. B. Snapper , D. S. Shouval , and J. A. Goettel . 2020. “Humanized Mouse Models of Genetic Immune Disorders and Hematological Malignancies.” Biochemical Pharmacology 174: 113671. 10.1016/j.bcp.2019.113671. 31634456 PMC7050416

[acel70313-bib-0063] Walsh, N. C. , L. L. Kenney , S. Jangalwe , et al. 2017. “Humanized Mouse Models of Clinical Disease.” Annual Review of Pathology 12: 187–215. 10.1146/annurev-pathol-052016-100332. PMC5280554 27959627

[acel70313-bib-0064] Weitzmann, M. N. , and R. Pacifici . 2006. “Estrogen Deficiency and Bone Loss: An Inflammatory Tale.” Journal of Clinical Investigation 116, no. 5: 1186–1194. 10.1172/JCI28550. 16670759 PMC1451218

[acel70313-bib-0065] Xia, X. , H. Li , S. Satheesan , J. Zhou , and J. J. Rossi . 2019. “Humanized NOD/SCID/IL2rγnull (Hu‐NSG) Mouse Model for HIV Replication and Latency Studies.” Journal of Visualized Experiments 143: 58255. 10.3791/58255. PMC6559246 30663638

[acel70313-bib-0066] Yang, Y. , J. Li , D. Li , W. Zhou , F. Yan , and W. Wang . 2024. “Humanized Mouse Models: A Valuable Platform for Preclinical Evaluation of Human Cancer.” Biotechnology and Bioengineering 121, no. 3: 835–852. 10.1002/bit.28618. 38151887

[acel70313-bib-0067] Yu, S. , F. Huang , Y. Huang , et al. 2024. “Deciphering the Influence of Gut and Oral Microbiomes on Menopause for Healthy Aging.” Journal of Genetics and Genomics 52, no. 5: 601–614. 10.1016/j.jgg.2024.11.010. 39577767

[acel70313-bib-0068] Zhao, H. , X. Li , D. Zhang , et al. 2018. “Integrative Bone Metabolomics—Lipidomics Strategy for Pathological Mechanism of Postmenopausal Osteoporosis Mouse Model.” Scientific Reports 8, no. 1: 16456. 10.1038/s41598-018-34574-6. 30405156 PMC6220250

[acel70313-bib-0069] Ziemian, S. N. , O. O. Ayobami , A. M. Rooney , et al. 2021. “Low Bone Mass Resulting From Impaired Estrogen Signaling in Bone Increases Severity of Load‐Induced Osteoarthritis in Female Mice.” Bone 152: 116071. 10.1016/j.bone.2021.116071. 34171515 PMC8863567

[acel70313-bib-0070] Zivkovic, A. M. , N. Telis , J. B. German , and B. D. Hammock . 2011. “Dietary Omega‐3 Fatty Acids Aid in the Modulation of Inflammation and Metabolic Health.” Calif Agric (Berkeley) 65, no. 3: 106–111. 10.3733/ca.v065n03p106. 24860193 PMC4030645

[acel70313-bib-0071] Zukić, M. , I. Taljić , and I. Banjari . 2024. “Effectiveness of Commercial Red Clover (*Trifolium pratense* L.) Products for the Treatment of Symptoms in Menopausal Women—A Narrative Review.” Nutraceuticals 4, no. 3: 430–449. 10.3390/nutraceuticals4030026.

